# Evaluation of *NTHL1*, *NEIL1*, *NEIL2*, *MPG*, *TDG*, *UNG *and *SMUG1 *genes in familial colorectal cancer predisposition

**DOI:** 10.1186/1471-2407-6-243

**Published:** 2006-10-09

**Authors:** Peter Broderick, Tina Bagratuni, Jairam Vijayakrishnan, Steven Lubbe, Ian Chandler, Richard S Houlston

**Affiliations:** 1 Section of Cancer Genetics, Brookes Lawley Building, Institute of Cancer Research, 15 Cotswold Road, Sutton, Surrey, SM2 5NG, UK

## Abstract

**Background:**

The observation that germline mutations in the oxidative DNA damage repair gene *MUTYH *cause colorectal cancer (CRC) provides strong evidence that dysregulation of the base excision repair (BER) pathway influences disease susceptibility. It is conceivable that germline sequence variation in other BER pathway genes such as *NTHL1*, *NEIL1*, *NEIL2*, *MPG*, *TDG*, *UNG *and *SMUG1 *also contribute to CRC susceptibility.

**Methods:**

To evaluate whether sequence variants of *NTHL1*, *NEIL1*, *NEIL2*, *MPG*, *TDG*, *UNG *and *SMUG1 *genes might act as CRC susceptibility alleles, we screened the coding sequence and intron-exon boundaries of these genes in 94 familial CRC cases in which involvement of known genes had been excluded.

**Results:**

Three novel missense variants were identified *NEIL2 *C367A, *TDG3 *A196G and *UNG2 *C262T in patients, which were not observed in 188 healthy control DNAs.

**Conclusion:**

We detected novel germline alterations in *NEIL2, TDG *and *UNG *patients with CRC. The results suggest a limited role for *NTHL1*, *NEIL1*, *NEIL2*, *MPG*, *TDG*, *UNG *and *SMUG1 *in development of CRC.

## Background

A recent twin study indicates that approximately a third of all colorectal cancers (CRC) involve an inherited predisposition [1]. Germline mutations in the known CRC genes (*APC*, mismatch repair (MMR) genes, *MUTYH/MYH*, *SMAD4*, *ALK3 *and *STK11/LKB1*) do not, however, account for all of the familial risk of the disease. The observation that mutations in *MUTYH *predispose to CRC [2,3]has provided strong evidence that dysregulation of the base excision repair (BER) pathway contributes to disease susceptibility. MUTYH functions as a DNA glycosylase responsible for excision of adenines mis-incorporated opposite 8-oxo-7,8-dihydro-2'-deoxyguanosine (8-oxoG), a stable product of oxidative DNA damage [4]. The BER pathway plays a pivotal role in protecting against oxidative DNA damage and is especially relevant in colorectum, which is characterised by high levels of oxygen radicals generated by bacteria and dietary carcinogens [5,6].

In addition to MUTYH a number of other DNA glycosylases participate in BER. These include endonuclease III-like 1 (*NTHL1*, MIM 602556) which acts on oxidized pyrimidine residues; endonuclease VIII-like 1 (*NEIL1*, MIM 608844) and endonuclease VIII-like 2, (*NEIL2*, MIM 608933) which initiate the first step in BER by cleaving reactive oxygen species (ROS) damaged bases; N-methylpurine DNA glycosylase (*MPG*; MIM 156565) which removes a diverse group of damaged bases, including cytotoxic and mutagenic alkylation adducts of purine; thymine-DNA glycosylase (*TDG*, MIM 601423) which initiates repair of G/T and G/U mismatches, commonly associated with CpG islands, by removing thymine and uracil moieties; uracil-DNA glycosylase (*UNG*, MIM 191525) which removes uracil in DNA resulting from deamination of cytosine or replicative incorporation of dUMP, and single-strand-selective monofunctional uracil-DNA glycosylase 1 (*SMUG1*; MIM 607753) which removes uracil from single- and double-stranded DNA in nuclear chromatin.

To evaluate whether germline variants of *NTHL1*, *NEIL1*, *NEIL2*, *MPG*, *TDG*, *UNG *and *SMUG1 *genes might act as CRC susceptibility alleles, we have screened the coding sequence and intron-exon boundaries of these genes in 94 familial CRC cases.

## Methods

### Ascertainment of cases and controls

Study subjects were ascertained as part of the National Study of Colorectal Cancer (NSCCG). Details of the NSCCG study design are available online [7,8]. Briefly, the NSCCG was established in March 1999 and is an ongoing study to investigate the role of genetic factors in the aetiology of CRC. To date over 6,000 cases with histologically verified adenocarcinoma of the colon or rectum have been recruited from clinics throughout the United Kingdom. A standardised questionnaire was used to collect phenotypic and family history information from cases and all were asked to provide blood samples for the extraction of DNA. The current study is based on CRC cases that reported family history of CRC in at least one first-degree relative. The 94 cases with the earliest age of CRC were selected. No cases carried biallelic *MUTYH *mutations or a truncating mutation in *APC *(associated with familial adenomatous polyposis). Germline mismatch repair gene mutations were excluded by microsatellite instability testing (BAT25, BAT26) in archival tumour specimens. Although not totally comprehensive it provides a relatively robust method of excluding inherited MMR mutations. Controls were cancer free individuals who were spouses or friends of cancer cases selected to match the sex and age of the cases as closely as possible. All study subjects were Caucasian of British ancestry and current UK residents. Genomic DNA was extracted and quantified from the venous blood samples by standard methods.

Informed consent was obtained from all participants and the study was undertaken with local ethical board approval in accordance with the tenets of the Helsinki Declaration.

### Mutational analysis

The coding regions and intron-exon boundaries of *NTHL1*, *NEIL1*, *NEIL2*, *MPG*, *TDG*, *UNG *and *SMUG1 *were PCR amplified. Amplifying primers were designed using the genomic sequence for each gene [9] in conjunction with Primer3 software [10]. Primer sequences and PCR conditions for each gene are detailed in Table 3. PCR products were hybridised and heteroduplexes assayed for small intragenic mutations by conformation sensitive gel electrophoresis (CSGE) [11]. Genomic DNA from cases showing mobility shifts was sequenced using the BigDyeTerminator Cycle Sequencingkit and a 3730xl automated sequencer (Applied Biosystems, Foster City, USA). All mutations were confirmed by sequencing at least two independent PCR products.

### Bio-informatics analysis

We applied two *in silico *algorithms, the PolyPhen algorithm [12,13] and the SIFT algorithm [14,15], to predict the putative impact of missense variants on protein function.

## Results

DNAs from the 94 familial CRC without germline mutations in the known CRC predisposition loci were screened for sequence variants in *NTHL1*, *NEIL1*, *NEIL2*, *MPG*, *TDG*, *UNG *and *SMUG1*. The average age at diagnosis of CRC in the cases was 54.8 years (SD, 9.3 years; median age 56 years). Of the cases 59 had been diagnosed with colonic disease and 54 were male.

In total 22 sequence variants were identified (Table 2). Eleven of the variants were detected in intronic (not involving consensus splice sites) or untranslated regions. One variant, rs5745908 maps to the second base of the donor splice site in intron 1 of *NEIL1*. Of these twelve variants, ten have been previously reported as polymorphisms (sequence variants with minor allele frequency greater or equal to 1%) in the dbSNP database [16]. On the basis of the likely absence of effect on protein function and the fact that most were seen in multiple cases, we consider it unlikely that these intronic variants represent high-risk CRC cancer susceptibility alleles. Therefore, these were not additionally investigated. Ten exonic variants were identified. None of the variants caused translational frameshifts or nonsense codons and three were synonymous (i.e. maintaining the amino acid sequence of the translated protein; Table 2). Of the three synonymous variants, two were known polymorphisms documented in dbSNP and only one was a novel change. Seven non-synonymous changes were identified and of these four were documented in dbSNP as polymorphisms. To investigate the population frequency of the three novel missense alterations identified in single unrelated cases (*NEIL2 *C367A, *TDG3 *A196G and *UNG2 *C262T) the relevant exons were screened in 188 cancer-free controls. None of these variants were detected in the controls. The three novel missense variants detected in familial CRC cases were predicted to be probably damaging by both the Polyphen and SIFT algorithms. *NEIL2 *C367A was detected in a 65-year-old male with CRC. The individual's brother, sister and nephew had also been diagnosed with CRC. Variant *TDG3 *A196G was identified in a 66-year-old male with rectal cancer. The patient's sister died of CRC at age 47. Variant *UNG2 *C262T was identified in a male with colonic cancer diagnosed at age 53 whose mother and maternal aunt had CRC diagnosed at age 77 and 72 respectively. Unfortunately for reasons of clinical governance we were not in a position to evaluate tumour blocks from relatives to test for segregation of alleles.

## Discussion

We have sought to identify pathogenic germline mutations in seven genes encoding components of the BER system. To empower our analysis we have studied familial cases in which involvement of the known CRC predisposition genes has been excluded. In ascertaining familial cases we have relied on reported information. While inaccuracy in reported family histories is a theoretic limitation, studies have shown that cancers such as CRC are generally reliably reported in first-degree relatives [17].

While MYH associated polyposis is an autosomal recessive disease we purposely did not restrict our analysis to individuals with these phenotypes as there is no evidence *a priori *that mutations in other BER will operate in a similar fashion, hence it is appropriate to consider all models of inheritance. None of the patients studied harboured clearly pathogenic biallelic sequence variants, nor was there strong evidence that any single variant was disease causing.

We cannot exclude the possibility that a minority of mutations have been missed, but under test conditions we have found that CSGE can detect all small insertions or deletions and 70% of single base substitutions. Here the technique detected a number of single base substitution polymorphisms hence there is over a 90% probability that an allele conferring a 2-fold increase in CRC risk with a population frequency = 1% will have been identified through screening the 94 familial cases. Based on the number of patients we have screened for constitutive mutations we can conclude with 95% probability that germline variation in any of these genes will at best not account for more than 3% of all familial CRCs in the British population (upper 95% confidence interval of point estimate).

We detected a number of novel germline alterations in *NEIL2, TDG, UNG *genes in patients with CRC. Three novel missense variants detected in familial CRC cases were predicted to be probably damaging by both the Polyphen and SIFT algorithms. While these algorithms have been demonstrated in benchmarking studies to successfully categorise 80% of amino acid substitutions [18], predictions about the functional consequences of amino acid changes are not definitive and require validation in functional assays. Overall results suggest at best a limited role for these variants in predisposition.

The substrates of the BER glycosylases overlap and therefore there is functional redundancy within the oxidative DNA damage repair system. On this basis it is perhaps not surprising that we did not identify disease causing mutations in our study. Such an assertion does not however, take into account the fact that mutation of *MUTYH *is causative of CRC. Finally, our current analyses do not exclude the possibility that sequence variants in the genes we analysed are associated with low penetrance CRC susceptibility. Evaluation of this hypothesis will require additional studies comparing the frequency of gene sequence variants in large series of CRC cancer cases and healthy controls.

## Conclusion

We report here the first *NEIL2, TDG, UNG *germline alterations in patients with CRC. However, the rarity of such alterations suggests a limited role for sequence variation in defining predisposition. Notwithstanding, germline variants in these genes do exist and may be associated with susceptibility, but further studies including functional analyses are needed for confirmation.

## Abbreviations

APC, adenomatosis polyposis coli; BER, base excision repair; CRC, colorectal cancer; CSGE, conformation sensitive gel electrophoresis; MMR, mismatch repair; MPG, N-@methylpurine DNA glycosylase; MUTYH, MutY, E.coli, homolog of; NEIL1, endonuclease VIII-like 1; NEIL2, endonuclease VIII-like 2; NSCCG, National Study of Colorectal Cancer; NTHL1, endonuclease III-like 1; ROS, reactive oxygen species; SMUG1, single-strand-selective monofunctional uracil-dna glycosylase 1; TDG, thymine-DNA glycosylase; UNG, uracil-DNA glycosylase

## Competing interests

The author(s) declare that they have no competing interests.

## Authors' contributions

PB, TB, JV, SL and IC carried out the genetic studies. PB and RSH were responsible for drafting and revising the manuscript. PB and RSH were involved in design of the study, providing important intellectual content and acquisition of the study material. All authors read and approved the final manuscript.

## Pre-publication history

The pre-publication history for this paper can be accessed here:



## Figures and Tables

**Table 1:** *NTHL1*, *NEIL1*, *NEIL2*, *MPG*, *TDG*, *UNG *and *SMUG1 *gnes screened for germline mutations.

**Gene**	**Genimic sequence**	**cDNA sequence**	**No. of exons**	**No. of PCR fragements**
*NTHL1*	NT_037887	NM_002528.4	6	7
*NEIL1*	NT_010194	NM_024608.1	9	11
*NEIL2*	NT_077531	NM_145043.1	4	6
*MPG*	NT_037887	NM_002434.2	4	8
*TDG*	NT_019546	NM_003211.3	10	12
*UNG*	NT_009775	NM_080911.1	7	9
*SMUG1*	NT_029419	NM_014311.1	2	5

**Table 2:** Sequence changes in BER genes in familial colorectal cancer cases and controls

**Gene **	**Exon**	**Nucleotide change**	**Amino acid change**	**No. of heterozygote cases (n = 94)**	**No. of heterozygote controls (n = 188)**	**DbSNP database entry**
*NEIL1*	IVS1	T434+2C		1		rs5745908
						
*NEIL2*	IVS1	T138+25C		>10		rs804269
	IVS1	C138+35T		>10		rs804268
	2	G308A	R103Q	1		rs8191613
	2	C367A	P123T	1	0	
	3	A564G		>10		rs8191642
	IVS3	C689-13T		>10		rs8191663
	4	G770T	R257L	1		rs8191664
	3' UTR	C999+21T		>10		rs1534862
	3'UTR	999+34 delC		>10		rs8191667
						
*MPG*	5' UTR	1–27 insT		1		rs3176380
	2	C147G		3		rs2259275
	3	C342G		1		
						
*TDG*	IVS2	A166+12G		4		rs3829300
	IVS2	G167-9A		>10		rs3751209
	IVS2	G167-19C		1		
	3	A196G	R66G	1	0	
	10	G1099 A	V367M	>10		rs2888805
						
*UNG*	2	C262T	R88C	1	0	
	2	G246C		0	1	
	IVS4	533–25		>10		rs3219235
	IVS6	801+20 delTTTT		>10		
						
SMUG1	1	G44T	G15V	1		rs2233920

**Table 3:** Sequences of primers and PCR conditions used to screen BER genes.

		**Primer sequence (5' > 3')**			
**Gene**	**Fragment**	**Forward**	**Reverse**	**Amplicon (bp)**	PCR Conditions*
***NTHL1***	1	GTAGTTCTGTGCCGCCCTCT	CAGCCTGCAGCCCCTATC	270	68–60 TD, 1.7M B
	2A	ATGAACGAGTGAGGGGTGAG	AGCTGTTGCTGCCAGTCC	244	68–60 TD, 1.7M B
	2B	AGAGACTGCGTGTGGCCTAT	GAGGGTGCCAGCCAAAAG	284	68–60 TD, 1M B
	3	CAGGGAATCACCCAGGAC	AGGTCTCTCTCAGGCCACTG	279	68–60 TD, 1M B
	4	GCTGCATCCTCCCAGGTT	CACAGGTCACAAGGATGTGG	298	68–60 TD
	5	GGCTAGGCTGGTGGAGTGT	GGGTCAGTGCTGACAGAGG	225	68–60 TD
	6	GGCTTCCTAGGGAGAAGAGC	GTGGCTTCCTGAAGCGTAAA	266	68–60 TD
					
***NEIL1***	1A	CAGCCGCTACCTCACAAAGT	GCTGAAGCTGAGATGCGGTA	232	68–60 TD
	1B	CAGCCAGTTTGTGAATGAGG	CCGTGTAAAAGCGCAGGT	281	68–60 TD
	1C	CCACTGGCCCTGGTCTTC	TTGGCCAAGAAGGCACTAAG	295	68–60 TD, 1M B
	2	AGGTTCTCTGAGCCCCTCTC	GCTAGGCAGTGGGGAATGT	263	68–60 TD
	3	GTGCCCACATTCCCCACT	CTAAGCTGGGGACACCTGAC	208	68–60 TD
	4	CCCAAAGTCTGACCAAGCTC	CCCCAGGGTTACACAATCAT	245	68–60 TD
	5	GAGCCTGCCCTCTGATCTCT	GGGGTCTCTGCCTGTGTG	203	68–60 TD
	6	CACTGATCTGGATGGGTGTG	ATAGGGTGGAGAAGGGTGCT	210	60–50 TD, 1M B
	7	AGCACCCTTCTCCACCCTAT	CCGCCCTCTTAGGGTAAGG	238	68–60 TD
	8	CCAACCTCTGAACTGCTTTCT	TAGAGCCAGTTTCTGGGAGTG	257	68–60 TD
	9	CGTGTACAAAGGTGGGAGAAA	AATTCAGACCCCCAGATGC	216	68–60 TD
					
***NEIL2***	1	TGCTTGGCACCTGTTAAAGA	CCCTGAGACATATGGGGATAGA	296	68–60 TD, 1M B
	2A	AATATCCGCATTCCCCAGTT	GGGCGTCTCTCTCCAAATAC	296	68–60 TD, 1M B
	2B	GGGCAGAAGACCCTTGATG	TGACTATCAGAGCCCACGAA	254	68–60 TD, 1M B
	3	CAGCCACAGGGTGTGCTCT	TCTGTGGACTCACCCCTTTC	299	68–60 TD, 1M B
	4A	GCCATGTGTCCTTTGTCCTT	GCCTCTGTAACCCATCTTCG	290	68–60 TD, 1M B
	4B	CGCAGCACACACAGGTCTAC	CCACATCCTCCTTTCTGGAC	237	68–60 TD, 1M B
					
***MPG***	1	GGTCCTAGGGGTGCTTCC	TGGCCTATGCTCTGGCTCT	254	68–60 TD, 1.7M B
	2A	GCTGTCTCCTATTCGGATGC	CCCTTTGGGCTTGAGAAATA	248	68–60 TD, 1.7M B
	2B	CACACAGCTCGTCCGATG	CAGTGACAGGCACTTCAGGA	229	68–60 TD, 1M B
	3A	TGGGCACTGTTAGGGTGAGT	ACCCTGGCTGGAGATGTTC	273	68–60 TD, 1M B
	3B	CTAGTCCGGCGACTTCCTAA	CCCCACCTCAGTCCTCCTA	291	68–60 TD, 1M B
	4A	GCAGAGAGGACAGGAGCCTA	CCAGCCATACAGCTTCATCC	267	68–60 TD, 1M B
	4B	GAGACCATGCGTCAGCTTC	ACATAGAAGCGGAGGGGTTT	266	68–60 TD, 1M B
	4C	GATGAAGCTGTATGGCTGGAG	GCTCTGGCTAAGGCACAGTT	270	68–60 TD, 1M B
					
***TDG***	1	TCTCTGGGGTTGTCTTACCG	AGCCTGCCCAGCAGTGAG	180	68–60 TD, 1M B
	2	TGATCATTTGGATTTACATTTGG	GGCTGATCCGATGTTGAACT	252	68–60 TD, 1M B
	3A	TTTTCTGGGAAAGCTGCTAAA	GGGGAGAGTCTTGGTCAGAA	279	60–50 TD
	3B	CTGGCAAGTCTGCAAAATCA	GGTCCTTTTCAGCAAAATGC	252	60–50 TD
	4	GATGAAATGTCTAATTGTTTTGTTTT	CACACAGAACATGAAACACGA	233	60–50 TD
	5	GGCTGCACTGAGCCATGAT	GGTTCCAACCCATAAAAGCA	271	68–50 TD
	6	TCAAGCTGAGCTCAACAAATG	CAAACATATTTACATTGCCCATAA	262	68–60 TD
	7	TCAGCCACGAATAGCAGTGT	TCACAATGGATAGGACAAATAAGG	288	68–60 TD
	8A	CAAGTTATTAACCCAAATAAAGACAAA	TGAATAAAAGGAATGAGGACAGTAA	300	60–50 TD, 1M B
	8B	CATCCAGTGCAAGATGTGCT	CACAAAATGAATAAAAGGAATGAGG	190	68–60 TD, 1M B
	9	CAAGAAAAAGAATTGTTCATGATTTC	CCTGACCAAACCGTCTTTGA	267	68–60 TD
	10	GGCGATAGAGTAAGACCCAGTC	GGTTCTACTTGTTGACAACTGCAT	298	68–60 TD
					
***UNG***	1	AATTGCTGACCGCCACAG	CCTTCCTCCCCCTTCACC	262	68–60 TD, 1M B
	2A	GGGCTCTTACTGTCCGCTTT	CTCTGGATCCGGTCCAACT	240	68–60 TD, 1M B
	2B	GACCACTTGCAGGCCATC	GGCCGGCTACACTAACAAGA	296	68–60 TD, 1M B
	3	TTGAATTCTTATGGTTTCCAATGA	TGTGGCTTAACTCCAGTGTCC	240	68–60 TD, 1M B
	4	CAGGGTCTGTGCTGCTTACA	AACAGTGCCCCAGATAGTCC	251	68–60 TD, 1M B
	5A	CAAGGGCTGGCTGTAACTTC	TAGCAGTCGCTGGCTTACCT	218	68–60 TD, 1M B
	5B	GGCTTGCTTTCAGTTTGGAG	CAGCTATGGTGGCTCATGC	288	68–60 TD, 1M B
	6	TGCCTGAGCCTACATTTAACC	GCAGGGACTCCTAGAATTCTTTA	299	60–50 TD, 1M B
	7	CCACTGCAGCAAGACTCTGT	GGAACTTCGTAACTGGCAAA		60–50 TD
					
***SMUG1***	1A	CTCTGTGGCTGAGGGTTGAT	TTGTAGATGATGCCCACAGG	233	68–50 TD, 1M B
	1B	AGAGCTTCCTGGAGGAGGAG	AGCCAAGCATCCACCTAGAA	258	68–60 TD, 1M B
	2A	TCTCCAGTTTGAAGCCTTTCAT	CTCAGCAGGAGTAAGGTTGC	300	68–60 TD, 1M B
	2B	CCACAATCTATGCCCTCTGC	TCAATCTTTCCTTGGCCACT	296	68–60 TD, 1M B
	2C	TCTTGGGATCTGTGATGCAG	TTCGAGGTCTTGAATGTGTCC	286	68–60 TD, 1M B

* TD, Touchdown °C; B, Betaine
